# Sociospatial structure explains marked variation in brucellosis seroprevalence in an Alpine ibex population

**DOI:** 10.1038/s41598-017-15803-w

**Published:** 2017-11-15

**Authors:** Pascal Marchand, Pauline Freycon, Jean-Philippe Herbaux, Yvette Game, Carole Toïgo, Emmanuelle Gilot-Fromont, Sophie Rossi, Jean Hars

**Affiliations:** 1 Office National de la Chasse et de la Faune Sauvage, Unité Ongulés Sauvages, Les Portes du soleil, 147 avenue de Lodève, F-34990 Juvignac, France; 2 UMR CNRS 5558 Laboratoire de Biométrie et Biologie Evolutive, VetAgro Sup, 1 Avenue Bourgelat, F-69280 Marcy L’Etoile, France; 3 Office National de la Chasse et de la Faune Sauvage, Service Départemental de la Haute-Savoie, 90 route du col de Leschaux, F-74320, Sévrier, France; 4 Laboratoire départemental d’analyses vétérinaires de la Savoie, 321 chemin des moulins, F-73000 Chambéry, France; 5 Office National de la Chasse et de la Faune Sauvage, Unité Sanitaire de la Faune, Micropolis - La Bérardie, 05000 Gap, France; 6 Office National de la Chasse et de la Faune Sauvage, Unité Sanitaire de la Faune, 5 allée de Béthléem, F-38610 Gières, France

## Abstract

In a context of (re)emerging infectious diseases with wildlife reservoirs, understanding how animal ecology shapes epidemiology is a key issue, particularly in wild ungulates that share pathogens with domestic herbivores and have similar food requirements. For the first time in Europe, brucellosis (*Brucella melitensis*), a virulent zoonosis, persisted in an Alpine ibex (*Capra ibex*) population and was transmitted to cattle and humans. To better understand disease dynamics, we investigated the relationships between the spatial ecology of ibex and the epidemiology of brucellosis. Combining home range overlap between 37 GPS-collared individuals and visual observations of 148 visually-marked individuals monitored during the 2013–2016 period, we showed that females were spatially segregated in at least 4 units all year round, whereas males were more prone to move between female units, in particular during the rutting period. In addition to ibex age, the spatial structure in females largely contributed to variation in seroprevalence in the whole population. These results suggest that non-sexual routes are the most likely pathways of intraspecific transmission, crucial information for management. Accounting for wildlife spatial ecology was hence decisive in improving our ability to better understand this health challenge involving a wildlife reservoir.

## Introduction

Wild animals have been identified as key components in the (re)emergence and worldwide spread of disease^1–3^. The numerical and spatial expansion of some species (for example, wild herbivores;^4^), along with the development of transport facilities and changes in agricultural systems (e.g. increasing size of domestic herds and decreasing human presence;^5,6^) have resulted in increased contact rates between wildlife and livestock^7^ and in the formation of disease reservoirs in wildlife^2^. Consequently, identifying the drivers of animal and disease distributions has become a key issue in preventing the transmission of infections between wildlife and domestic herds and the subsequent formation of reservoirs in wildlife^8^.

In this context, large herbivores have been recognized as particularly relevant species for studying disease processes in free-ranging populations and for understanding how wildlife-livestock interactions may affect such processes^9^. Indeed, wild and domestic herbivores share similar food requirements, foraging behaviours and pathogens, increasing the risks of interspecific transmission of contagious diseases. In addition, the spatial ecology (e.g. area-restricted space use, partial migration) and social organization (e.g. fission-fusion dynamics, spatial and/or sexual segregation) of wild herbivores give rise to complex host-to-host interactions (intra- or interspecific) and make disease dynamics in these species particularly tricky (see^10^ for a review of large herbivore ecology). Hence it is increasingly important to better understand how the ecology of large herbivores can shape disease epidemiology.

The spread of disease through a population and transmission between sympatric species are generally related to when and how individuals come into contact, either by direct interactions or by the habitat they share^11,12^. In social species, several characteristics such as group size, mating systems, segregation and fission-fusion dynamics have also been revealed as key factors determining intra- and interspecific epidemic dynamics^13–16^. For example, the social organization in badger *Meles meles* populations from south-west England has been shown to be of prime importance in driving bovine tuberculosis dynamics. As a result, the drastic culling that was a central part of attempts to control the disease could in practice increase movements between social units and hence the spread of disease^17^. The relationships between the spatial behaviour of wildlife, socio-spatial organizations and the spread of epidemics have consequently become a key research theme in disease ecology^8,15^.

We focused on the first report of brucellosis (*Brucella melitensis*) transmission from wildlife to domestic ruminants and humans, which was revealed in 2012 in the northern French Alps (Bargy massif; 46°00′N, 6°28′E, 1500–2438 m above sea level)^18,19^. Two human cases due to the virulent *Brucella melitensis* biovar3 were detected and related to the consumption of cheese made from fresh raw milk from an infected cattle herd grazing in this area^20^. Given that a previous outbreak involving a very similar *Brucella* strain had occurred in 1999 in livestock (sheep and cattle) in the same area and that all other 205 domestic herds that grazed in the massif from 1999 up to 2012 were found brucellosis-free before they were taken to alpine pastures, wildlife was suspected of being the source of disease persistence and re-emergence. Preliminary analyses confirmed the persistence of this virulent zoonotic pathogen in a wild ruminant species^18,19^. The local population of Alpine ibex (*Capra ibex*), a protected species of high patrimonial value, was heavily infected. Seroprevalence reached 38% in 2013 (95% CI [28.2; 47.8]; n = 77) and *B. melitensis* was isolated from several organs and lymph nodes of the first necropsied individuals suggesting high excretion levels^21,22^. Other large herbivores inhabiting the same area had a much lower rate of infection (chamois [*Rupicapra rupicapra*]; 1.8%, n = 55) or were not infected (roe deer [*Capreolus capreolus*], n = 44 and red deer [*Cervus elaphus*], n = 30). In an attempt to control the wildlife reservoir, several management actions were undertaken by the French Authorities between 2013 and 2015, including an important ibex slaughtering program: 251 individuals over 5 years old, representing > 44% of the estimated population, were culled in October 2013 and May 2014 (see Methods).

Previous brucellosis outbreaks had been reported in Iberian and Alpine wild ruminants (chamois, isard [*Rupicapra pyreneica*] and ibex), as self-limiting events, so that these species were generally considered dead-end hosts^23–26^. However, this persisting outbreak poses serious threats for public health and for the regional economy, as it occurs in a mountain agricultural area with an important production of a famous soft raw milk cheese (reblochon; agriculture revenues reaching € 20 millions in 2011). Indeed, brucellosis is a barrier to trade in animals and animal products. It causes significant losses from abortions, that generally occur during the last third of gestation^27^, and because the whole domestic herd has to be slaughtered when one individual is infected^28^. In domestic ruminants, brucellosis can be transmitted (i) from mother to young (so-called vertical transmission), (ii) by the sexual route during mating, or by the horizontal route, i.e. (iii) direct contacts between two individuals or (iv) indirect interactions between one individual and the infected secretion/abortion products of another through a contaminated environment^28^. This latter transmission route may be particularly important in disease spread as abortion products are heavily infected, resulting in several individuals being contaminated at once when they are aggregated and/or share the same environment. This has been confirmed in several domestic ruminants^28,29^ and already highlighted in wild species at the interface between bison (*Bison bison*) and elk (*Cervus canadensis nelsoni*) in the Greater Yellowstone Ecosystem (*Brucella abortus* ^30^). By contrast, little is known on how brucellosis *Brucella melitensis* could have persisted in that ibex population. Hence, the important public health, economical, conservation and management concerns posed by this brucellosis outbreak justified deep investigations on the relationships between the ecology of Alpine ibex and the epidemiology of brucellosis in this population.

Alpine ibex generally use distinct seasonal home ranges (migratory individuals) but can also restrict space use to a single annual home range (sedentary individuals) depending on individual and/or habitat characteristics^31,32^. Preliminary information on space use by ibex from the Bargy massif suggested limited seasonal migration, both in terms of the proportion of individuals concerned and of distance some individuals travelled between summer and winter ranges^33^. The use of a single annual home range, along with the importance of social bonds in this gregarious species, may result in the existence of spatially-separated units that use exclusive ranges most of the year. Such “spatial segregation” has been observed in several ibex populations^32,34–37^. Within each spatial unit, strong sexual segregation, strengthened with male age and with both social and habitat/ecological components, was reported outside the rutting period^34,38,39^. As a result of different foraging needs, activity patterns, social affinities and strategies to ensure reproductive success, males and females generally live in distinct groups that do not use habitats in the same way or at the same time^40^. The social component of this sexual segregation may predominate in spring, when groups of males and groups of females share first snow-cleared and developing pastures^34^. Conversely, the habitat/ecological components contribute the most to sexual segregation during summer, when females select steep areas perceived as safe to give birth and raise their offspring, while males focus on food-rich habitats^40,41^. Segregation based on both social and habitat components was also reported between males of different ages^34,37^. Sexual segregation dropped during the rutting period, however, when roving males seeking females display high movement capabilities increasing connectivity between spatially-segregated units^42,43^.

As both spatial and sexual segregations have been previously reported in the Bargy massif^34,35,37^, we hypothesized that spatial and social organizations could shape transmission opportunities among the ibex population studied, resulting in variation in brucellosis prevalence among spatial units and among age-sex classes. To test this hypothesis, we first combined home range overlap between 37 GPS-collared individuals with visual observations of 148 marked individuals, to check the existence of several spatially-segregated units within the studied population. We then assessed the extent to which the spatial structure we identified, in addition to age (related to the duration of brucellosis exposure, and to the level of segregation between sexes and between males), was related to spatial variation in brucellosis seroprevalence. Finally, we also assessed the influence of the slaughtering program conducted in October 2013 and May 2014 on seroprevalence variation and on space use of individuals monitored during common periods before and after these operations. By coupling information on ibex space use, socio-spatial organization, and disease prevalence in the population, we were aiming for a better understanding of brucellosis dynamics and intraspecific transmission routes in this complex health and economic challenge involving wildlife.

## Results

**Figure 1:**
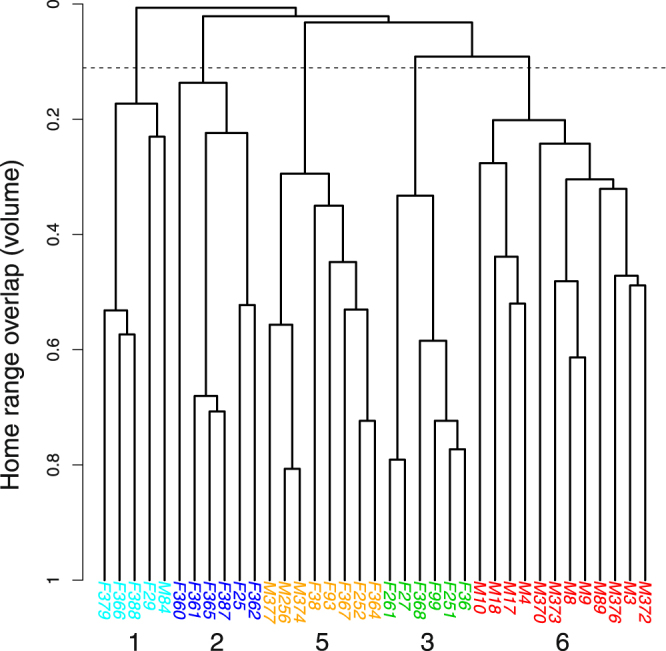
Hierarchical classification tree representing the structure in 37 GPS-collared Alpine ibex (*Capra ibex*) from the Bargy massif based on overlap between annual home ranges as a measure of distance between individuals.

**Figure 2:**
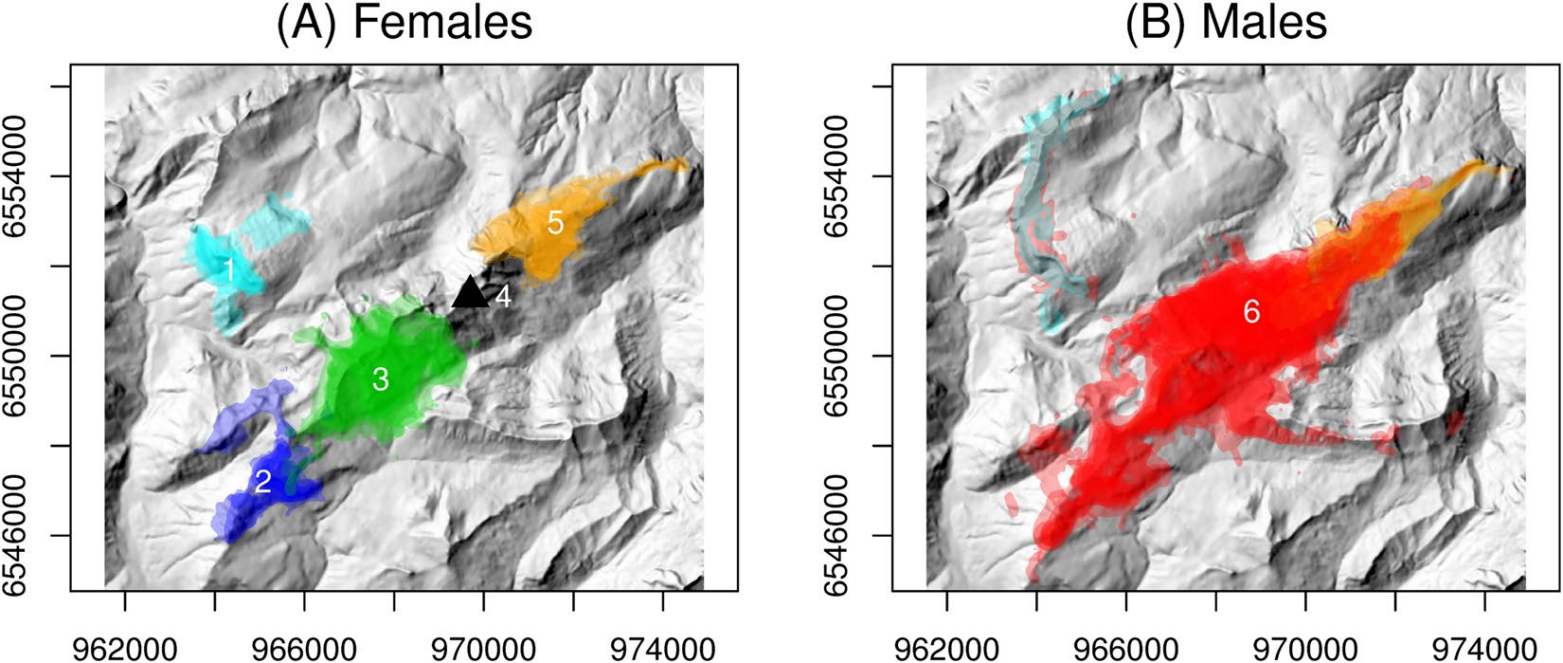
Annual home ranges of GPS-collared Alpine ibex (*Capra ibex*) (**A**) females (n=21) and (**B**) males (n=16) from the Bargy massif (northern French Alps). The colors correspond to the different units identified using overlap between annual home ranges as a measure of distance between individuals (see Fig. 1). The black triangle represent the area where we did not equip any female with GPS collars but where some ibex were captured or slaughtered. These maps were created using R version 3.4.1^80^: https://cran.r-project.org.

In the Bargy massif, five ibex units could be distinguished among the 37 GPS-collared ibex (Fig. 1). Four included females only or females and males (#2, #3, and #1, #5 in Fig. 1, respectively; see below for the definition of unit #4) whereas the last one consisted of males only (#6). The representation of annual home ranges of each individual on sex-specific maps revealed that females belonging to different units were indeed spatially-segregated, whereas the home ranges of females from the same unit largely overlapped in restricted areas (Fig. 2A). By contrast, males from unit #6 intensively used a large area occupied by several female units (unit #3 and #5), but also visited the areas used by units #1 and #2 and consequently overlapped most of the female ranges (Fig. 2B). The classification trees provided by the overlap between seasonal home ranges confirmed that the spatial structure in females was mostly stable all year round (Supplementary information 1). By contrast, males from unit #6 were more prone to move in the whole study area except during summer and autumn, when they used a restricted area located between the ranges of units #2 and #5, and hence largely overlapped with females from unit #3 (Supplementary information 2 and Fig. 2).

Observations recorded each year from May to September of the 2013–2015 period confirmed that the 148 marked individuals followed the same spatial structure as that identified in the 37 GPS-collared individuals, and that capture location provided reliable information on the unit to which each individual ibex could be assigned (Supplementary information 2). This was particularly true for females: they were generally observed in the range used by GPS-collared individuals from the spatial unit to which they were assigned based on capture locations (511/530 observations, 96.4%). By contrast, visual observations confirmed males were more prone to move between these spatial units, with only 54.5% (415/762) of observations falling in the spatial range used by individuals from the spatial unit to which they were assigned.

**Figure 3:**
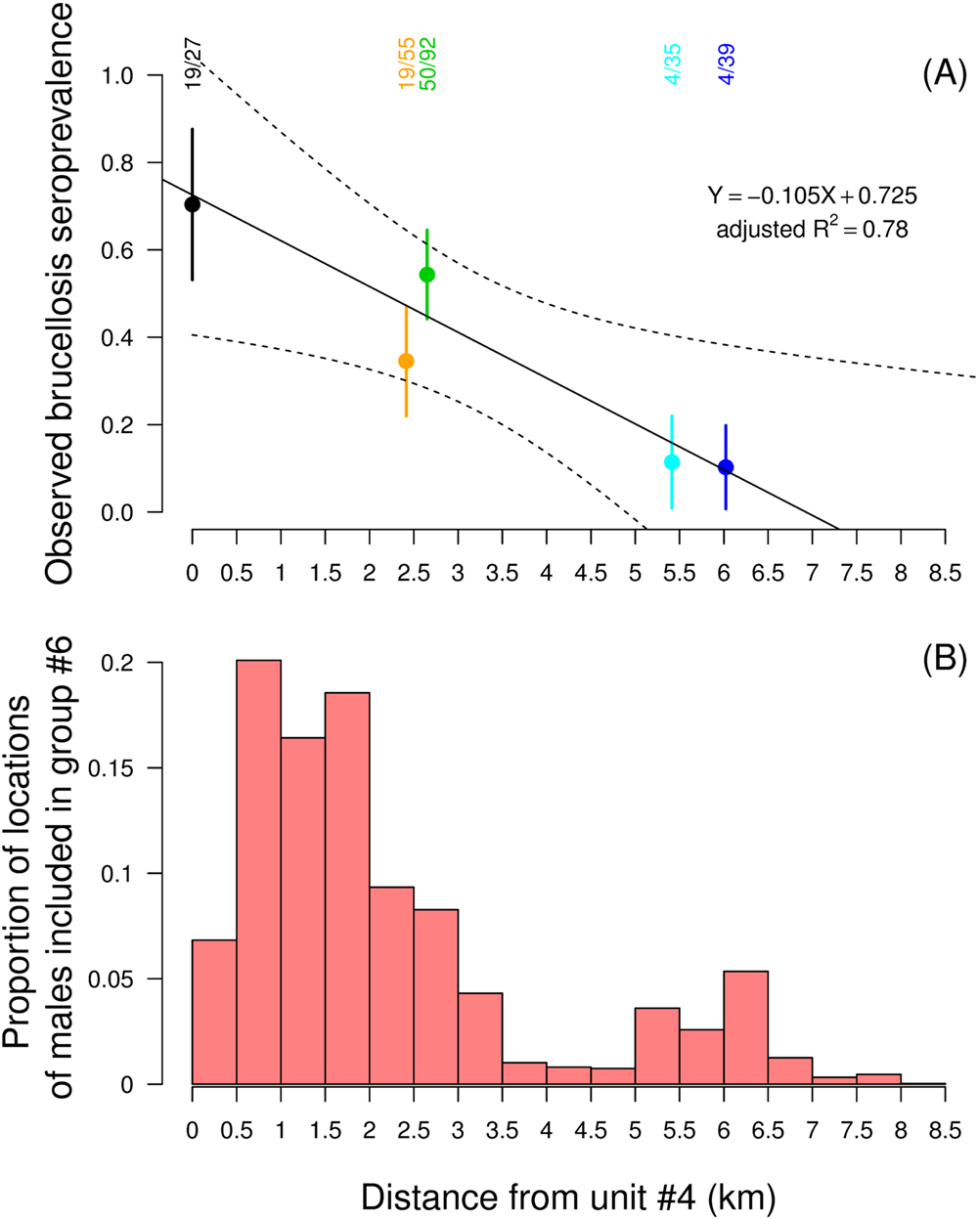
(**A**) Variation in the observed brucellosis seroprevalence in the identified sociospatial units of the Alpine ibex (*Capra ibex*) population from the Bargy massif and (**B**) Distribution of locations from males included in unit #6 according to distance from the highly infected unit #4. In panel (A), the solid line and the dashed lines represent predictions and the 95% confidence interval, respectively, from the weighted linear regression accounting for the number of individuals tested for brucellosis in each unit and which parameters are given on the right side. The raw numbers of seropositive versus tested Alpine ibex in each unit are given above.

Since some ibex were captured or slaughtered in an area that was not used by the females equipped with GPS collars (#4, black triangle in Fig. 2A), we included data on seroprevalence collected in this area as an undetermined group in the modelling procedure. In addition, given that females were spatially-structured all year round, and that a similar structure could be identified in males during some seasons, but that males from unit #6 largely overlapped with females from units #3 and #4 and other males (see Figs 1, 2A and 3), we tested for several classifications for males when investigating the influence of this spatial structure on brucellosis seroprevalence: (i) each individual male, including those from unit #6, was assigned to the female unit that used the area in which it was captured (“unitsMFall” in Supplementary information 3), (ii) males from units #3 and #4 were considered a unique group (corresponding to unit #6 in Figs 1 and 2B]) and those from the other units were assigned to the female unit that used the area in which each individual was captured (“unitsMF” in Supplementary information 3), and (iii) all the males were assigned to a unique group not spatially structured (“unitsF” in Supplementary information 3). Only the results provided by the classification that best explained the seroprevalence variation in the massif (i.e. “unitsMFall” in Supplementary information 3), are presented below.

**Figure 4:**
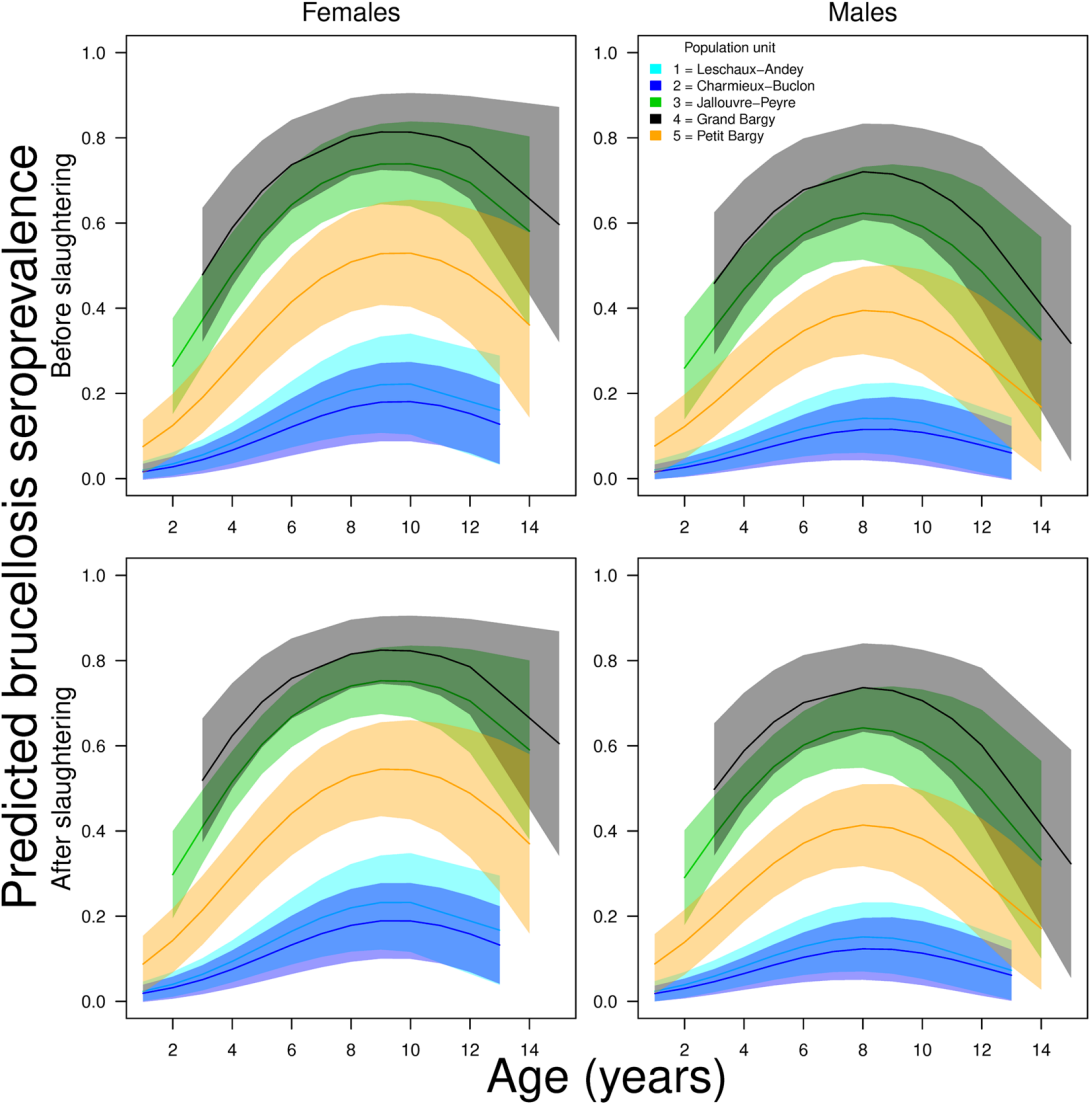
Predictions from the model-averaging procedure investigating the influence of the sociospatial structure identified, sex, age (quadratic term) and slaughtering operations on brucellosis (*Brucella melitensis*) seroprevalence in the Alpine ibex (*Capra ibex*) from the Bargy massif (northern French Alps; see Table 1 for details on the set of candidate models). The colors correspond to the different units identified using overlap between annual home ranges as a measure of distance between individuals (see Fig. 1).

Among the multiple factors included in a logistic regression approach to explain variation in the serological status of 246 Alpine ibex individuals, the spatial structure identified in females had a strong influence on brucellosis seroprevalence in the population as a whole (i.e. including males; “unitsMFall” in Table 1), in addition to age (quadratic term, “age” in Table 1). This was revealed by the high relative importance (>0.99) and the persistent presence of both parameters in the top-ranked models (Table 1 and Supplementary information 4). Coefficients provided by the model-averaging procedure (Table 2) and the representation of the predictions from the model-averaging procedure (Fig. 4) highlighted in both sexes clear differences between units #1 and #2, where seroprevalence was <*30*% (raw data: 4/39 and 4/35 positive individuals, i.e. 11.4% and 10.3%), and units #3 and #4, where seroprevalence was significantly higher and could reach a maximum of 80% depending on ibex age (50/92 and 19/27, i.e. 54.3% and 70.4%). Seroprevalence values recorded in unit #5 were intermediate (<50%; raw data: 19/55, i.e. 34.5%). However, seroprevalence rose strongly during the first years of life, reached a plateau around 8–10 years in both sexes, and decreased in older individuals, in particular in males. In addition, there was a strong decrease in brucellosis seroprevalence with increasing distance between the centroid of each population unit and the centroid of the highly infected unit #4 (weighted linear model, p = 0.030, *β* = −10.5%.km^−1^, R^2^ = 0.78; Fig. 3A). Similarly, the proportion of locations of males included in unit #6 largely decreased with increasing distance from unit #4 (Fig. 3B).

**Table 1:** Selection of models fitted to investigate variation in brucellosis seroprevalence in the Alpine ibex *Capra ibex* population from the Bargy massif (northern French Alps). Only the models with AIC_*c*_ weight ≥ 0.05 are provided here; for the full list of candidate models, see Supplementary Information 4. In model acronyms, “ + ” corresponds to additive effects and “×” to the interaction between the 2 factors. *k* is the number of parameters, LL is the maximum log-likelihood, ΔAIC*c* is the difference in the Akaike information criterion between the model with the lowest AIC_c_ and the other models, and AIC_c_ weight is Akaike weight. “Age” and “sex” are ibex age (quadratic term) and sex, respectively. “UnitsMFall” are sociospatial population units (see Fig. 1 and Supplementary information 1). “Periods” opposed data collected before and after the slaughtering operations that occurred during autumn 2013 and early spring 2014.

Model	k	LL	AIC_*c*_	ΔAIC_*c*_	AIC_*c*_ weight
unitsMFall + sex x age	10	−129.53	279.99	0.00	0.27
sex + age + unitsMFall	8	−131.84	280.28	0.29	0.23
age + unitsMFall	7	−133.17	280.82	0.83	0.18
unitsMFall + period + sex x age	11	−129.17	281.47	1.47	0.13
unitsMFall + age + period	8	−132.66	281.94	1.94	0.10

**Table 2:** Coefficients provided by the model-averaging procedure investigating the influence of the sociospatial structure identified (i.e. unit #1 to #5), sex, age (quadratic term) and slaughtering operations on brucellosis (*Brucella melitensis*) seroprevalence in the Alpine ibex (*Capra ibex*) from the Bargy massif (northern French Alps; see Table 1 for details on the set of candidate models). In parameter acronyms, “×” corresponds to the interactive effect between both parameters. *β* is the estimated value, *SE* is the standard error of the estimated value, *95% CI* is the 95% confidence interval. The reference group (intercept) corresponds to females from unit #2 before the slaughtering operations.

Parameter	*β*	*SE*	95% *CI*	p-value
intercept	−2.32	0.63	[−3.56; −1.09]	0.00
males	−0.49	0.35	[−1.11; 0.13]	0.33
age	9.04	4.66	[0.06; 18.02]	0.06
age^2^	−6.19	3.37	[−12.70; 0.33]	0.07
unit #4	3.03	0.75	[1.57; 4.50]	0.00
unit #3	2.59	0.64	[1.34; 3.83]	0.00
unit #5	1.65	0.66	[0.36; 2.94]	0.01
unit #1	0.24	30.48	[−59.49; 59.97]	0.99
age × males	−10.70	6.22	[−21.11; −0.30]	0.49
age ^2^ × males	−1.64	3.47	[−12.10; 8.83]	0.85
after slaughtering	0.38	0.28	[−0.40; 1.15]	0.68
age × after slaughtering	−9.05	2.98	[−22.28; 4.17]	0.82
age ^2^ × after slaughtering	2.08	1.72	[−9.76; 13.92]	0.93
age × unit #4	−31.14	1.89	[−94.67; 32.40]	0.98
age × unit #3	−34.73	1.97	[−96.24; 26.77]	0.97
age × unit #5	−36.78	2.03	[−98.58; 25.03]	0.97
age × unit #1	−35.23	2.15	[−107.94; 37.48]	0.98
age ^2^ × unit #4	10.12	0.95	[−29.60; 49.84]	0.98
age ^2^ × unit #3	14.45	1.01	[−23.46; 52.37]	0.98
age ^2^ × unit #5	6.52	0.87	[−32.19; 45.23]	0.99
age ^2^ × unit #1	−18.41	1.86	[−97.15; 60.33]	0.99
after slaughtering × unit #4	1.61	0.07	[−1.40; 4.62]	0.98
after slaughtering × unit #3	1.28	0.06	[−1.42; 3.97]	0.98
after slaughtering × unit #5	−0.49	0.04	[−3.19; 2.21]	0.99
after slaughtering × unit #1	15.02	30.47	[−1914.88; 1944.92]	1.00
males × unit #4	−0.27	0.02	[−3.23; 2.69]	1.00
males × unit #3	1.04	0.03	[−1.48; 3.56]	0.99
males × unit #5	0.81	0.02	[−1.82; 3.43]	0.99
males × unit #1	1.87	0.04	[−1.30; 5.04]	0.99
males × after slaughtering	−0.15	0.00	[−1.44; 1.14]	1.00

By contrast, ibex gender was less important in explaining variation in brucellosis seroprevalence (“sex”; relative importance = 0.38, i.e. 2.6 times less important than spatial struture and age) even though the interaction between age and gender was included in the model with the lowest AIC_*c*_. We also detected no difference in brucellosis seroprevalence between individuals tested before (n = 54) or after (n = 192) the massive slaughtering program conducted in 2013–2014 (“periods” in Table 1, relative importance = 0.31, i.e. 3.2 times less than the spatial structure and age; Fig. 4). The average overlap between the ranges used by GPS-collared individuals monitored during common periods before and after the slaughtering operations reached 0.61 in females (SD = 0.04; range = [0.56; 0.66]; n = 4) and 0.52 in males (SD = 0.17; range = [0.26;0.74]; n = 8), i.e. values comparable with those that previously allowed including different individuals in the same spatial unit (Fig. 1 and Supplementary information 1). This result suggested that the slaughtering operations had only limited consequences on individual ibex space use in the short term (1–2 years).

## Discussion

Combining space use by 37 GPS-collared individuals and visual observations of 148 marked individuals, we identified that ibex females of the Bargy massif were spatially structured, whereas males were more prone to move between the exclusive ranges used by each female unit. This spatial structure in females, in addition to age, explained the high variation in brucellosis seroprevalence in the population as a whole. By contrast, once spatial structure and age were accounted for, seroprevalence was similar in both sexes and did not change significantly after slaughtering operations. Such results provide crucial information to understanding brucellosis dynamics and identifying the major intraspecific transmission routes, key information to design management strategies for this exceptional outbreak of disease.

Our analyses confirmed that ibex females from the Bargy massif were spatially segregated in at least four units with little or no overlap all year round. Since the definition of these spatial units was based on a limited number of individuals (i.e. 21 females and 16 males) monitored with GPS collars, the existence of one or more additional unit(s) could not be excluded. However, visual observations confirmed the existence of this spatial structure in 148 marked individuals, particularly in females, and suggested it could be extrapolated to the whole population (Supplementary information 2). Besides, the existence of such a spatial structure had already been proposed in this population though it had not been accurately defined^34,37^. As in many other gregarious herbivore species with philopatric females (e.g.^44,45^, in Mediterranean mouflon,^46^ in chamois and isard [*Rupicapra pyrenaica*],^47^ in bighorn sheep [*Ovis canadensis*]), social constraints and area-restricted space use resulted in marked spatial segregation between female units^36,37^.

This sociospatial structure was the most determining factor influencing brucellosis seroprevalence, a result consistent with reports made in other epidemiologic contexts involving large herbivores^13,14^. For example, spatial segregation accounted for marked differences in the prevalence of chronic wasting disease in a mule deer (*Odocoileus hemionus*) population from Colorado^48^. It is also a key factor explaining behavioral, phenotypic, genetic and/or fitness variations in populations of large herbivores^46,49–51^. Previous analyses that did not consider this spatial structure found contrasting seroprevalence levels in individuals between 2 and 5 years old before/after slaughtering operations^33^. However, the sampling effort for these individuals was evenly distributed in spring 2012–spring 2013 (22/33 individuals tested in units #3, #4 and #5) but mostly focused on highly-infected units in spring 2014 (37/39 individuals in units #3, #4 and #5), probably explaining why this period effect was no longer detected here. These results suggest that the absolute number of animals was less determinant in disease transmission than the spatial distribution of females in the massif. However, the persistence of this spatial structure after slaughtering operations and the consequences on brucellosis seroprevalence could also be questioned. Indeed, changes in movements and space use, and social reorganizations could be expected after intense culling and removal of 40% of the population (see^52^ and^53^ for examples in perturbed populations of badgers and lions [*Panthera leo*]), respectively). Currently, the high overlap between the utilization distribution of GPS-collared ibex monitored before and after the slaughtering operations, and data on seroprevalence (see Results section) suggested limited consequences on space use and disease dynamics in the short term (1–2 years). Despite this apparent stability of the spatial structure and of seroprevalence levels over the study period, focusing on the dynamics of the social network structuring this population^54^, and of seroprevalence over the years, could in the future allow us to determine if social reorganizations had occurred, and evaluate their epidemiological consequences. It could also allow assessing the relative contribution of spatial segregation between population units, and social and habitat segregation within them, in the variation of the brucellosis seroprevalence we reported here^34,37–39^.

Interestingly, the structure revealed in females was also a key determinant of the seroprevalence observed in males, although males move frequently among spatial units, in particular males from unit #6 (Fig. 3, Supplementary information 1 and 2). The heterogeneous prevalence among spatial units despite frequent movements of males suggest that direct and indirect transmission due to females, in particular via the environmental contamination within each spatial unit, is probably more important for intraspecific transmission than the sexual and vertical modes^28^. Indeed, since males move among female spatial units, particularly during the rutting period, a purely sexual transmission would lead to spatial homogenization of brucellosis seroprevalence. However, movements of males and sexual transmission should not be excluded as a potential route of disease spread between units. Indeed, the drop in seroprevalence levels with increasing distance from the most infected unit #4, and the similar pattern in the distribution of locations of males included in unit #6 (Fig. 3), suggest the individuals using this area were of preeminent importance in the maintenance and spread of disease towards other units through source-sink dynamics^55,56^. As a purely speculative scenario, one could suggest that an important infectious event (e.g. one or several abortion[s]) may have occurred in this area (used by units #4, #3 and #6), infecting many individuals at the same time. During this outbreak, seroprevalence may have exceeded the level below which brucellosis disappeared naturally during previous *B. melitensis* outbreaks involving wild ruminants^23–26^. Since then, infection may have spread from the highly infected area to other units, essentially due to the movements of males from unit #6. A similar scenario, with behavioral connectivity among spatially-segregated units depending mostly on movements of males, has been recently proposed to explain pneumonia spread in bighorn sheep populations^47^. Nevertheless, the causes of such a hypothetic massive initial infectious event, and the mechanisms driving the exceptional persistence of this spatial pattern over time, are difficult to determine. Differences in ibex density, group size and structure between units, possibly related to landscape heterogeneity driving ibex aggregation, still have to be investigated.

Mother-to-offspring transmission, that may also occur in some infected but still fertile females (as in sheep and goats^28^), could be one of the mechanisms contributing to the exceptional persistence of the spatial pattern over time, since mother and offspring live in the same areas for a couple of years. However, infection may pass undetected before the reproductive maturity of young individuals, i.e. before 3–5 years in Alpine ibex^57,58^. Indeed, brucellosis can establish latent infection in sexually immature individuals and be undetectable by serological tests until the first reproductive event^59–61^). The increase in seroprevalence with age observed in young ibex of both sexes may reflect both their cumulative exposure throughout life and the increasing detectability of brucellosis with age. Likewise, the seroprevalence drop observed in old individuals, and particularly old males, may be related to antibodies dropping below detectable levels, or to low survival rates in old individuals infected by brucellosis^62,63^. However, these hypotheses are currently impossible to test in our population given that few old individuals were captured and that seropositive individuals were all euthanized.

Further research is needed to better understand the causes of persistence and the dynamics of this exceptional brucellosis outbreak in a wild ruminant species previously considered a dead-end host. These analyses should first investigate individual variation in space use of males and their relative contribution to disease spread. Whereas some males seemed to follow the same spatial structure as females, others were more prone to move between these units. Focusing on spatio-temporal variation in contact rates within and between populations units may then constitute a reliable approach to identifying risky areas and periods for intraspecific disease transmission^64^. Particular attention should be paid to late winter and early spring, i.e. when abortion events may occur^27^ and when ibex aggregation on the first snow-cleared and developing pastures is frequent, with little sexual segregation^34^. As the abortion products may be heavily infected^28,29^ and as bacteria have the ability to survive and remain infectious to other animals several months outdoors in cold and wet conditions^28^, even indirect contacts during this period may be particularly important in disease dynamics. In the Greater Yellowstone Ecosystem, the spatial overlap between bison and elk peaked when late-abortion and parturition events occurred for bison, increasing the risk of brucellosis *Brucella abortus* interspecific transmission^30^. In our case, this is also the period when most captures occurred, probably explaining that capture location significantly structured seroprevalence in both females and males in our population, even though both genders segregate outside of the rutting period and do not have exactly similar space use and movements. Finally, a spatially-structured epidemiological model is essential to test our hypotheses/scenario and determine the most effective management alternatives (e.g. biosafety measures, vaccination, approaches limiting transmission from highly-infected to less-infected units, culling, and combinations of these operations) promoted by the non-uniform distribution of brucellosis seroprevalence in the ibex population, i.e. differentiated management plans in the areas used by the different units, for both ibex and domestic herds^8,65–67^. This could offer new ethically and economically acceptable opportunities to ensure public health and preserve agricultural economy while saving this population of conservation concerns. Accounting for wildlife spatial ecology was hence decisive in improving our ability to understand this current complex health challenge involving a wildlife reservoir.

## Methods

### Study area, brucellosis and ibex population monitoring

Like most Alpine ibex populations, the Bargy population originates from a reintroduction, i.e., 6 males and 8 females translocated from the Mont-Pleureur population (Switzerland) between 1974 and 1976 (D. Gauthier, personal communication^67^). Following several bottlenecks, the genetic diversity in this population is low but comparable to levels observed in other reintroduced ibex populations (E. Quéméré, personal communication^68^). Some censuses were irregularly conducted on this population between the 1980’s and the late 1990’s, but no data have been collected since then. As a result, population size, spatial range and individual movements, performance (survival, reproduction, biometry) and sanitary status were unknown in 2012, when brucellosis re-emerged. Between 2012 and 2015, we captured (sometimes several times) 247 individuals by dart-gun xylazine/ketamine anesthesia (Rompun®, Bayer, Leverkusen, Germany and Imalgène®, Merial, France; 100 mg/individual). We took blood samples from all of them and tested serum samples for diagnosis of brucellosis according to the requirements of the World Organisation for Animal Health in small ruminants. Serological assays detected antibodies using the Rose Bengal test, the complement fixation test, indirect ELISA (IDEXX, Montpellier, France) and competitive ELISA (Ingenasa, Madrid, Spain), and a rapid test in the field (Anigen Rapid G.S. Brucella Ab, Bionote, Gyeonggi-do, Republic of Korea^69,70^; since 2014). In the present study, the serological status of animals was considered positive when at least two tests gave positive results. Seropositive individuals were shot (2013) or euthanized by embutramide intravenous injection (T61®, Intervet, Angers, France, 2014 and 2015; total = 98 positive individuals removed) whereas 149 seronegative individuals were released^33^. We recorded the sex and the approximate capture location (i.e. capture site, defined based on topographical landmarks) of all ibex tested, and determined age by counting horn growth annuli^71^.

Among the released seronegative ibex, we fitted 21 females and 16 males (between 2 and 14 years-old, 85% ≥ 4 years old, hence sexually mature) with GPS collars (GPS Plus, Vectronic Aerospace, Berlin, Germany; 530 g for females, 720 g for males, representing ≤1.5% of their body weight) programmed to record animal position every hour for at least 6 months, including at least one period when Alpine ibex generally migrate (between mid-May and mid-November^31,32^, see Supplementary information 5 for details on individuals equipped and on the monitoring). We screened GPS data for positional outliers based on unlikely movement characteristics^72^.

In addition, we marked the 149 seronegative ibex released using ear tags and/or collars so that individuals could be identified from several hundred meters using binoculars or a telescope. During the snow-free seasons (May-September) of the 2013–2015 period, we then recorded the composition (sex/age classes) of all the ibex groups observed, identity of marked individuals and reported group locations on a 100 × 100 m grid. We recorded most of these observations during the censuses we performed once a month between May and August, during which 1–2 observers travelled on 1 of the 9 tracks in the morning of a single day or 2 consecutive days (to avoid double counting). Using these mark-resight data and an immigration-emigration logit-normal model^73^, the population size (without newborns) estimated during summer 2013 was 567 (CI 95% [487; 660]). It dropped to 310 [275; 352] in 2014 and 277 [220; 351] in summer 2015. In 4 years, 359 individuals were removed: some because seropositivity was detected upon captures (n = 98; see above) and others (n = 261) as the result of management decisions taken by the French authorities. These decisions consisted in slaughtering individuals observed with clinical signs (autumn 2012 [n = 2] and spring 2013 [n = 8], total = 10), individuals > 5 years old (October 2013 [n = 233] and May 2014 [n = 18], total = 251).

All the ibex captured have been treated by professionals from the Office National de la Chasse et de la Faune Sauvage according to the ethical conditions detailed in the specific accreditations delivered by the Préfecture de Paris (prefectural decree 2009–014), by the French Minister of Ecology, Sustainable Development and Energy (Ministerial Orders of February 11, 2014) and by the Préfecture de la Haute-Savoie (prefectural decree 2015062-0018) in accordance with the French environmental code (Art. R421-15 to 421-31 and R422-92 to 422-94-1). Euthanasia was performed by veterinarians in accordance with the requirements of these accrediting authorities, and slaughtering operations were performed in accordance with accreditations delivered by the Préfecture de la Haute-Savoie (prefectural decrees 2013274-0001 and DDT-2015-0513). We hence confirm that we treated animals in accordance with relevant guidelines and regulations and that our protocols were approved by these institutions.

### Ibex space use, overlap and spatial structure in GPS-collared ibex

Using hourly locations collected by GPS collars, we computed individual utilization distribution (UD) using the Biased Random Bridge approach (BRB^74^). This method is based on the biased random walk movement model. It takes into account animal’s movement path and time between locations to calculate UD, the probability density function providing likelihood of an animal occurring in each unit of a defined area, i.e. the 50 m cells of a raster grid, during the monitoring period. It supposes that animal movement is governed by a drift component (a general tendency to move in the direction of the next relocation) and a diffusion component (tendency to move in other directions than the direction of the drift). We determined the drift and diffusion components for each individual using the locations triplets collected over 3 hours and the maximum likelihood approach developed by Benhamou *et al*.^74^, respectively. We thus defined home range as the area including 95% of the space use estimated by the BRB approach.

We then used the overlap in volume between individual annual UD as a proxy of the distance and contact rates between individuals^75,76^. This dimensionless overlap index not only accounts for the area shared by two individuals but also for the relative use of this area by both individuals. It ranges from 0 (i.e. no spatial overlap) to 1 (i.e. 100% spatial overlap and identical use of this area by two individuals, or by one individual during two periods). We relied on a hierarchical classification procedure to investigate the existence of a spatial structure^77^ and on multiple methods to determine the best number of spatially-segregated groups in ibex monitored by GPS collars (see^78^ for details). We defined this best number of groups as the number provided by the majority of the methods tested. In order to evaluate the potential seasonal variation in this spatial structure, we also performed the same classification procedure using overlap between seasonal UD distinguishing spring (April-June), summer (July-August), autumn (September-09 November), the rutting period (10 November–14 January) and winter (15 January–March; Supplementary information 1).

In addition, we also checked whether visual observations of seronegative individuals marked with ear tags or collars confirmed the existence of the spatial structure we identified in the 37 GPS-collared individuals. Each individual was assigned to the closest unit based on the location where it was captured (possible for n = 148 individuals). We then computed the proportion of locations of individual assigned to the focal unit that actually fall within the range used by GPS-collared individuals from the same unit.

Finally, we also relied on the overlap in volume between the UD of GPS-collared individuals monitored during common periods before and after the slaughtering program to assess the influence of these operations on Alpine ibex space use.

### Relationships between spatial structure in GPS-collared ibex and brucellosis seroprevalence at the population scale

To test for the effect of the spatial structure we identified on seroprevalence variation in the whole ibex population, we first performed the same assignment to the different spatial units as for marked individuals. We assigned each individual tested for brucellosis for the first time (recaptures were removed from analyses) to the closest unit based on the location where it was captured or slaughtered. This assignment was possible for 246 individuals ranging from 1 to 15 years old, after removing ibex having obvious clinical signs, to avoid bias in prevalence estimation.

We then included the spatial structure identified in GPS-collared ibex in a modeling procedure aimed at investigating the drivers of brucellosis seroprevalence in the massif between 2012 and 2015. The serological status of each individual was coded as 1 (positive) or 0 (negative) and this response variable was analyzed using logistic regression (generalized linear models with a binomial error distribution and a logit link function). We tested for the influence of ibex sex, age as a continuous variable in years (linear and quadratic terms; for clarity, only results including quadratic terms, providing the best results, are presented). We also tested for a “period” effect, opposing data collected before and after the first slaughtering operations that occurred during autumn 2013 and spring 2014; this was done to test the hypothesis proposed earlier for potential consequences of slaughtering operations on brucellosis prevalence (by potentially modifying ibex space use and involving social reorganizations^33^). We compared all the models including the additive effects of these variables. We also tested for sex-specific variation in seroprevalence with age to account for the gradual age-specific segregation between males and females and between males of different ages^34,37,38^. In addition, given that a large majority of the individuals slaughtered were > 5 years old, we also tested for age-specific consequences of these operations by including an interactive effect between age and period. We based our model ordering on Akaike’s Information Criterion with second-order adjustment (AIC_*c*_). We considered the set of models for which the difference in AIC_*c*_ with the lowest AIC_*c*_ value (ΔAIC_*c*_) was <2 the best models^79^. In addition, we assessed the relative importance of each explanatory variable by summing the Akaike weights across all the models where each variable occurs^79^. Akaike weights can be interpreted as the probability that a model is the best model, given the data and the set of candidate models. Finally, we represented the sex- and age-specific variation in seroprevalence in the different spatial units predicted by a model-averaging procedure to account for uncertainty in model selection^79^.

We performed all analyses using R version 3.4.1^80^, and “adehabitatHR”, “adehabitatLT”^81^ packages for the computation of Utilization Distribution and overlap, and “AICcmodavg” for the model-averaging approach^82^.

### Data availability

The datasets generated during and/or analysed during the current study are available from the corresponding author on request to anyone who wishes to repeat our analyses or collaborate with us.

## Electronic supplementary material

Supplementary information
