# Supplementary material for: Sociospatial structure explains marked variation in brucellosis seroprevalence in an Alpine ibex population
Source: Sci Rep. 2017 Nov 15;7:15592. doi: 10.1038/s41598-017-15803-w (PMC5688143; doi:10.1038/s41598-017-15803-w)
Supplement: Supplementary file 1 — Supplementary information [file 41598_2017_15803_MOESM1_ESM.pdf]

Supplementary information

Sociospatial structure explains marked variation in brucellosis seroprevalence in an Alpine ibex population

Pascal MARCHAND, Pauline FREYCON, Jean-Philippe HERBAUX, Yvette GAME, Carole TOIGO, Emmanuelle GILOT-FROMONT, Sophie ROSSI, Jean HARS

Supplementary information 1

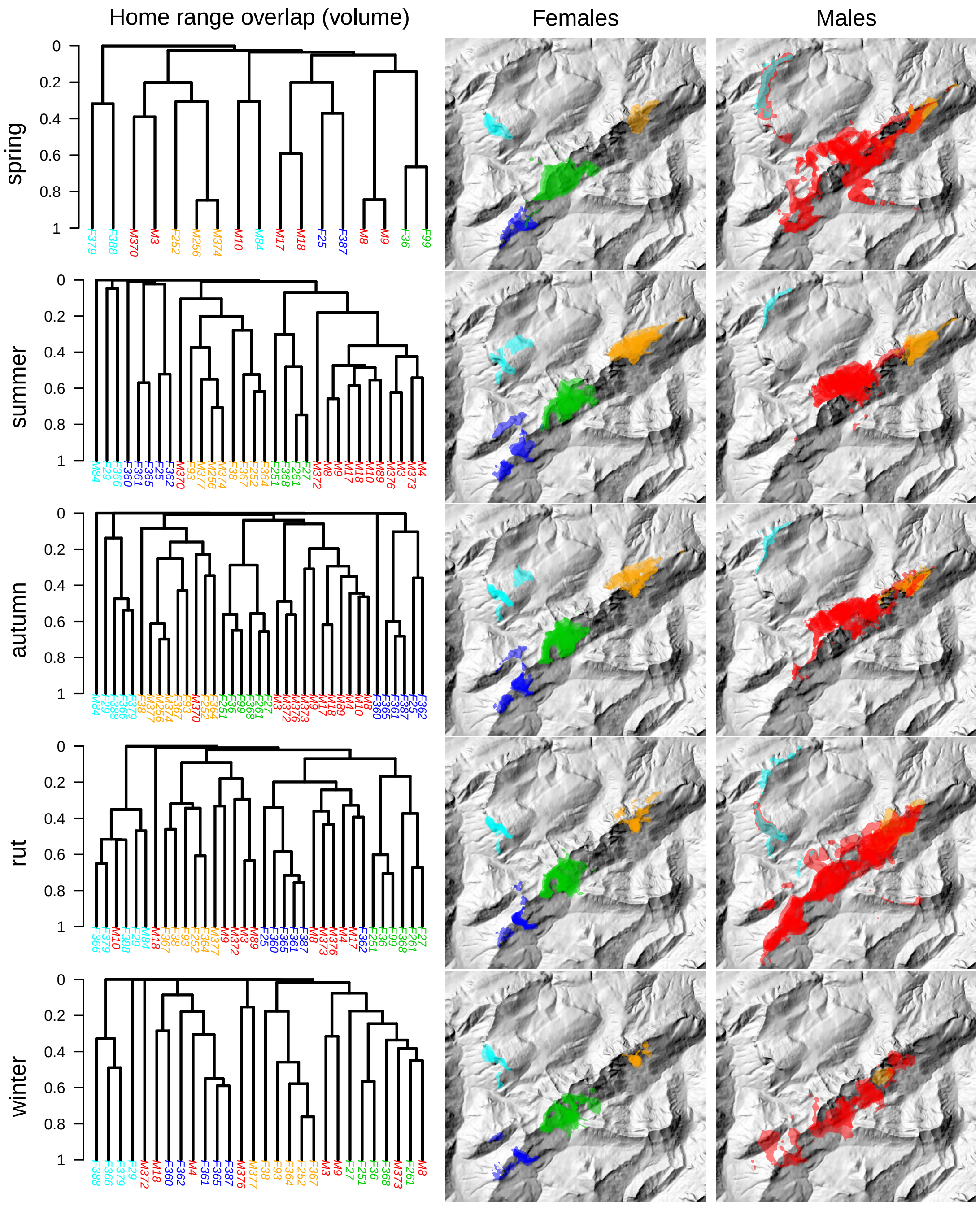

Seasonal variation in space use and spatial structure in 37 GPS-collared Alpine ibex (*Capra ibex*) from the Bargy massif based on overlap between seasonal home ranges as a measure of distance between individuals.
In order to evaluate the potential seasonal variation in the spatial structure we identified, we performed the same classification procedures as previously presented but using overlap between seasonal UD distinguishing spring (April-June), summer (July-August), autumn (September-09 November), the rutting period ("rut"; 10 November - 14 January) and winter (15 January - March). Only individuals monitored during at least half of the focal season are included in analyses. The corresponding home ranges are represented on sex-specific maps. Colors correspond to the different units identified using overlap between annual home ranges as a measure of distance between individuals (see Figure 1). These maps were created using R version 3.4.1[80]: https://cran.r-project.org.

Supplementary information 2

Do the locations of 148 visually-marked individuals confirm the spatial structure identified in the 37 GPS-collared individuals ?

To check whether the visual observations of the seronegative individuals marked with ear tags or collars allowing individual identification confirmed the existence of the spatial structure identified in the 37 GPS-collared individuals (see Figures 1 and 2 for details), we assigned each visually-marked individual to the closest spatial unit based on its capture location (possible for n=148 individuals; number of individuals per sex and spatial unit are given in the figure after "n=").

Then, for each spatial unit, we calculated the proportion of locations of individual assigned to a given unit that actually fell within the range used by GPS-collared females from the same unit (given in figure as "number of observations in the correct spatial range" / "total number of observations from individuals assigned to the focal unit" and as percentages).

We collected these locations in 2012-2015, during the snow-free season (May-September) by recording the composition (sex/age classes) of all the ibex groups observed, identity of marked individuals and by reporting group location on a 100×100m grid. We recorded most of these observations during the censuses that we repeated once a month between May and August, during which 1-2 observers travelled on 1 of the 9 tracks in the morning of a single day or 2 consecutive days.

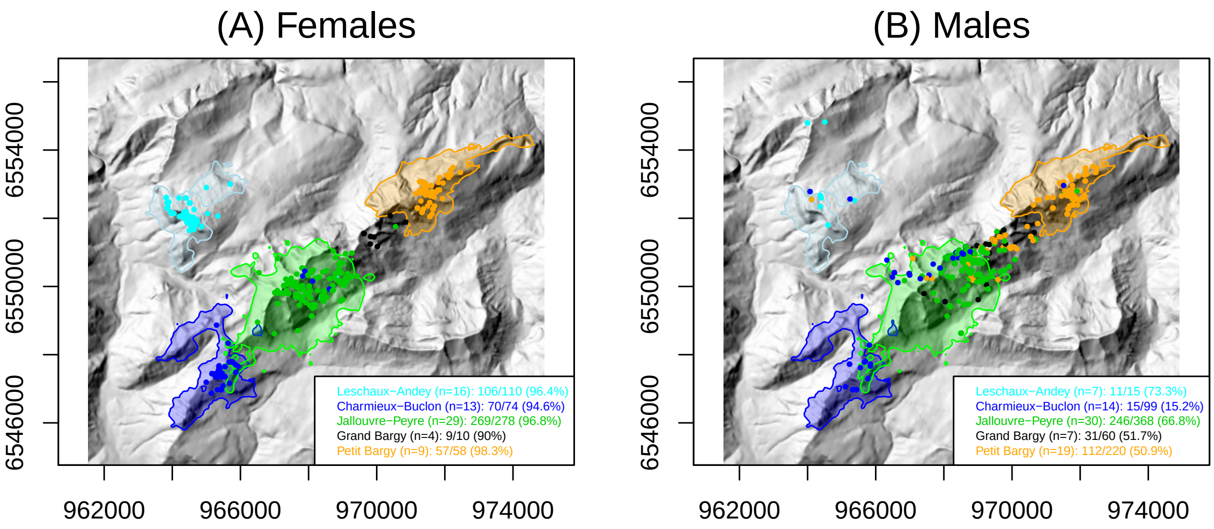

Locations of observations from visually-marked Alpine ibex (A=71 females; B=77 males) and ranges used by the GPS-collared females (n=21) from the corresponding population unit in the Bargy massif (French Alps).
These maps were created using R version 3.4.1[80]: `https://cran.r-project.org`.

Supplementary information 3

Model selection table investigating how several classifications of males affect the ordering of models investigating brucellosis seroprevalence in the Alpine ibex (*Capra ibex*) population from the Bargy massif (northern French Alps).

These models included either (i) the same sociospatial structure in both sexes (“unitsMFall”, i.e. individuals divided in units #1 to #5, same approach as that presented in the manuscript), (ii) a sex-specific sociospatial structure (“unitsMF”, i.e. females divided in units #1 to #5 and males divided in units #1, #2, #5 and #6 [unit #6 grouping males from units #3 and #4]), or (iii) a sociospatial structure only for females, males being considered a unique group not spatially structured (“unitsF”). In model acronyms, ”+” corresponds to additive effects and “$\times$” to the interaction between the 2 factors. k is the number of parameters, LL is the maximum log-likelihood, ΔAIC_c is the difference in the Akaike information criterion between the model with the lowest AIC_c and the other models, and AIC_c weight is Akaike weight. “Age” and “sex” are ibex age (quadratic term) and sex, respectively. “Periods” opposed data collected before and after the slaughtering operations that occurred during autumn 2013 and early spring 2014.

Model	k	LL	AIC_c	ΔAIC_c	AIC_c weight
sex x age + unitsMFall	10	-129.53	279.99	0.00	0.23
sex + age + unitsMFall	8	-131.84	280.28	0.29	0.20
sex + age + unitsMF	9	-131.15	281.07	1.08	0.13
sex + age + unitsMFall + period	9	-131.31	281.38	1.39	0.11
sex x age + unitsMFall + period	11	-129.17	281.47	1.47	0.11
sex x age + unitsMF + period	11	-129.21	281.54	1.55	0.10
sex + age + unitsMF + period	10	-130.66	282.25	2.25	0.07
sex + age + unitsF	8	-138.38	293.37	13.38	0.00
sex + age + unitsF + period	9	-137.69	294.14	14.15	0.00
age x unitsF	18	-131.15	301.31	21.31	0.00
age x unitsF + period	19	-130.60	302.57	22.58	0.00
sex x age + unitsMF	12	-128.87	283.08	3.08	0.05
null	1	-163.61	329.24	49.25	0.00

Supplementary information 4

Table 4.1. Candidate models fitted to investigate variation in brucellosis seroprevalence in the Alpine ibex *Capra ibex* population from the Bargy massif (northern French Alps). In model acronyms, ”+” corresponds to additive effects and “×” to the interaction between the 2 factors. *k* is the number of parameters, LL is the maximum log-likelihood, ΔAIC_c is the difference in the Akaike information criterion between the model with the lowest AIC_c and the other models, and AIC_c weight is Akaike weight. “Age” and “sex” are ibex age (quadratic term) and sex, respectively. “UnitsMFall” are sociospatial population units (see Figure 1 and Supplementary information 1). “Periods” opposed data collected before and after the slaughtering operations that occurred during autumn 2013 and early spring 2014.

Model	k	LL	AIC_c	ΔAIC_c	AIC_c weight
unitsMFall + sex x age	10	-129.53	279.99	0.00	0.27
sex + age + unitsMFall	8	-131.84	280.28	0.29	0.23
age + unitsMFall	7	-133.17	280.82	0.83	0.18
unitsMFall + period + sex x age	11	-129.17	281.47	1.47	0.13
unitsMFall + age + period	8	-132.66	281.94	1.94	0.10
sex + unitsMFall + age x period	11	-130.23	283.58	3.59	0.04
unitsMFall + age x period	10	-131.79	284.52	4.53	0.03
sex + unitsMFall	6	-138.18	288.72	8.72	0.00
unitsMFall	5	-139.38	289.01	9.01	0.00
sex + period + unitsMFall	7	-137.60	289.68	9.69	0.00
period + unitsMFall	6	-138.81	289.97	9.98	0.00
age x unitsMFall	15	-128.98	290.05	10.06	0.00
period x unitsMFall	10	-135.17	291.28	11.28	0.00
sex x unitsMFall	10	-136.56	294.05	14.06	0.00
sex + age	4	-155.63	319.43	39.44	0.00
age	3	-156.99	320.09	40.09	0.00
sex + age + period	5	-155.28	320.82	40.82	0.00
age + period	4	-156.59	321.35	41.36	0.00
sex x age	6	-155.02	322.39	42.39	0.00
sex + age x period	7	-154.05	322.58	42.58	0.00
age x period	6	-155.56	323.48	43.49	0.00
sex	2	-162.11	328.28	48.28	0.00
null	1	-163.61	329.24	49.25	0.00
sex + period	3	-161.81	329.72	49.73	0.00
period	2	-163.26	330.57	50.57	0.00
sex x period	4	-161.78	331.74	51.74	0.00

Supplementary information 5

General information on the Alpine ibex *Capra ibex* equipped with GPS collars in the Bargy massif (French Alps) between 2013 and 2016.

“Begin” and “End” are the dates the GPS monitoring began and ended, respectively. “Nb. relocations” is the total number of relocations recorded by GPS collars during the monitoring duration.

id	Sex	Age (years)	Begin	End	Duration (days)	Nb. relocations
F-25	F	11	2013-04-25	2014-01-01	250	5724
F-29	F	5	2013-05-10	2014-06-24	409	9313
F-36	F	4	2013-05-15	2015-07-02	777	16708
F-27	F	10	2013-05-23	2015-01-06	593	6985
F-38	F	5	2013-05-24	2014-05-06	346	7965
F-93	F	8	2013-06-06	2014-05-06	333	7545
F-99	F	2	2013-08-13	2016-02-12	913	10702
F-252	F	14	2014-06-20	2016-04-28	677	15075
F-261	F	2	2014-06-20	2016-01-09	568	6381
F-251	F	11	2014-06-27	2015-06-27	364	8417
F-366	F	4	2015-05-08	2016-04-11	338	7187
F-364	F	3	2015-05-12	2016-05-16	369	4238
F-368	F	2	2015-05-13	2016-05-02	354	7481
F-367	F	1	2015-05-18	2016-04-28	345	7900
F-361	F	10	2015-05-23	2016-04-15	327	7413
F-362	F	9	2015-05-28	2016-04-15	322	7212
F-365	F	6	2015-05-28	2016-04-21	328	7540
F-360	F	12	2015-06-03	2016-05-18	349	7637
F-387	F	8	2015-09-17	2016-05-29	255	2205
F-379	F	5	2015-09-19	2016-05-30	254	2219
F-388	F	4	2015-09-19	2016-05-30	254	1987
M-18	M	5	2013-04-27	2014-06-22	421	9732
M-17	M	5	2013-04-27	2014-11-24	576	10570
M-3	M	5	2013-04-30	2014-10-29	546	12572
M-10	M	8	2013-05-06	2014-02-08	278	6465
M-9	M	9	2013-05-09	2015-11-07	912	13921
M-8	M	6	2013-05-09	2015-12-02	937	12524
M-84	M	4	2013-05-10	2014-02-20	286	6466
M-89	M	11	2013-05-24	2015-01-28	614	8980
M-256	M	9	2015-05-07	2015-12-03	210	4165
M-374	M	11	2015-05-07	2015-11-13	190	4132
M-373	M	4	2015-05-07	2016-05-27	385	3206
M-370	M	8	2015-05-07	2015-12-12	219	2549
M-376	M	6	2015-05-08	2016-05-27	385	2825
M-372	M	9	2015-05-11	2016-05-07	361	9012
M-377	M	5	2015-05-12	2016-05-27	381	2088
M-4	M	7	2015-06-13	2016-05-30	351	3083
